# Association between ischemic heart disease and dietary intake of lycopene: a case–control study

**DOI:** 10.3389/fnut.2023.1281539

**Published:** 2024-01-08

**Authors:** Arezoo Amjadi, Farkhondeh Alami, Mohammad Keshavarz Mohammadian, Seyed Reza Mirshafaei, Fatemeh Azaryan, Anahita Houshiar-Rad, Mina Esmaeili, Soheila Shekari, Morteza Abdollahi, Sara Khoshdooz, Marjan Ajami, Saeid Doaei, Maryam Gholamalizadeh

**Affiliations:** ^1^Department of Nutrition, School of Nutritional Sciences and Food Technology, Kermanshah University of Medical Sciences, Kermanshah, Iran; ^2^Student Research Committee, Department of Nutrition, Faculty of Medicine, Urmia University of Medical Sciences, Urmia, Iran; ^3^Department of Nutrition, Science and Research Branch, Islamic Azad University, Tehran, Iran; ^4^Department of Applied Mathematics, Faculty of Mathematical Sciences, Roudsar and Amlash Branch, Islamic Azad University, Roudsar, Iran; ^5^Department of Physiology, School of Medicine, Zahedan University of Medical Sciences, Zahedan, Iran; ^6^Department of Nutrition Research, Faculty of Nutrition Sciences and Food Technology, National Nutrition and Food Technology Research Institute, Shahid Beheshti University of Medical Sciences, Tehran, Iran; ^7^Department of Nutrition Research, National Nutrition and Food Technology Research Institute, School of Nutrition Sciences and Food Technology, Shahid Beheshti University of Medical Sciences, Tehran, Iran; ^8^Department of Nutrition Research, National Nutrition and Food Technology Research Institute; and Faculty of Nutrition Sciences and Food Technology, Shahid Beheshti University of Medical Sciences, Tehran, Iran; ^9^Faculty of Medicine, Guilan University of Medical Sciences, Rasht, Iran; ^10^Department of Food and Nutrition Policy and Planning, National Nutrition and Food Technology Research Institute, School of Nutrition Sciences and Food Technology, Shahid Beheshti University of Medical Sciences, Tehran, Iran; ^11^Department of Community Nutrition, School of Nutrition and Food Sciences, Shahid Beheshti University of Medical Sciences, Tehran, Iran; ^12^Cancer Research Center, Shahid Beheshti University of Medical Sciences, Tehran, Iran

**Keywords:** ischemic heart disease, lycopene, dietary intake, coronary heart disease, IHD

## Abstract

**Aim:**

The effect of dietary lycopene on ischemic heart disease (IHD) is not clear. Hence, this study aimed to determine the association between dietary lycopene and IHD.

**Methods:**

This case–control study was conducted on 443 patients with physician confirmed diagnosis of IHD as the case group and 443 healthy individuals as the control group. Data on demographic, medical history, anthropometric, and physical activity of the participants were collected. Food intake was evaluated using a 237-item semi-quantitative food frequency questionnaire (FFQ). The dietary intake of lycopene was assessed using Nutritionist IV software.

**Results:**

A negative association was found between IHD and lycopene (OR: 0.98, CI 95%: 0.963–0.996, *p* = 0.02). The results remained significant after adjustment for age and sex, additional adjustment for dietary intake of calorie and fat, further adjustments for BMI, and additional adjustment for smoking, drinking alcohol, and physical activity. The risk of IHD in people with the highest quartile of dietary intake of lycopene was significantly lower than those with the lowest quartile (OR = 0.67, CI 95%: 0.46–0.97, *p* = 0.036).

**Conclusion:**

There was a significant inverse relationship between intake of lycopene and IHD. Further prospective studies in different populations are required to elucidate the roles of lycopene against IHD.

## Introduction

Ischemic heart disease (IHD) is a pathological condition characterized by decreased cardiac blood flow that causes a non-accordance between myocardial oxygen supply and demand (1). The number of patients diagnosed with IHD increased in recent years and the prevalence of IHD was reported to be around 126 million individuals (1,655 per 100,000), approximately 1.72% of the world’s population (2). The most common complications of IHD include acute mitral regurgitation (MR) secondary to papillary muscle rupture (PMR), ventricular septal defect (VSD), pseudoaneurysm, and free wall rupture (FWR). Each of these complications are related to increased risk of morbidity, mortality, and length of hospitalization (3, 4).

IHD has several risk factors such as genetic, socioeconomic factors, industrialization, urbanization, increased life expectancy, inadequate physical activity, and alternation of dietary patterns (5, 6). Numerous nutrients in fruits, vegetables, legumes, nuts, and seeds could be protective against IHD including potassium, dietary fibers, carotenoids, and subtypes of polyphenols (i.e., phenolic acids, flavonoids, stilbenes and lignans) (7–13). Lycopene is a member of the carotenoid family, a class of compounds found in fruits and vegetables (14–16). Growing evidence has indicated that lycopene’s antioxidant properties protect against cardiovascular disease, diabetes, and inflammatory diseases (17). Some studies suggested that lycopene’s antioxidant capabilities cause its cardioprotective effects. Also, Lycopene blocks angiotensin-converting enzyme (ACE) and may acts in reducing oxidative stress caused by angiotensin II and indirectly increasing NO synthesis in the endothelium (18).

Furthermore, Lycopene suppresses reactive oxygen species production, potentially preventing endothelial dysfunction through direct antioxidative actions (19). A recent meta-analysis found an inverse association between fruits and vegetables with risk of IHD (20). Furthermore, randomized controlled trials have shown that increased consumption of fruits and vegetables combinedly reduces blood pressure (21–23). In addition, Numerous studies have shown that higher intakes or blood concentrations of carotenoids have been linked to a reduced risk of CVD (13). A population-based study has shown that a lower risk for acute coronary events or stroke was associated with higher serum lycopene concentration (13). In agreement with the previous reports, the results of one nested case–control study demonstrated that higher plasma lycopene concentrations had been related to a lower risk of CVD in middle-aged and elderly women (24).

There are few studies on the association between lycopene and IHD (25–28). Moreover, the results of these studies have been inconsistent (29). Lycopene’s role has been ascribed to its potent antioxidant properties and other functions of lycopene such as gene expression regulation not yet completely understood. Many aspects regarding the roles of lycopene against IHD independent from other environmental and dietary factors are still unknown (13). The aim of the present case–control study was to evaluate the association between lycopene and the risk of of IHD after adjusting a broad range of confounders.

## Methods

The present case–control study was conducted on 443 patients with physician-confirmed IHD as the cases and 443 individuals without IHD as the controls. The sample size was obtained using Open EPI online software (30) and the odds ratio obtained in similar previous studies (28). A consecutive method was applied for selection of the case group among newly diagnosed subjects who were visited the Shahid Rajaei Hospital and Tehran Heart Center in Tehran, Iran. They all had IHD. Then, an oral explanation was given about the aim, the study’s implementation, and the information’s confidentiality. The control group was selected among individuals who visited the hospital for general check-up or were from the hospital staff without diagnosed heart disease. All demographic information, medical history, anthropometric measurements, physical activity levels, and food intake information were collected by a trained interviewer. The inclusion criteria for the case group were adults aged 40–80, suffering from IHD, diagnosed in the last three month before the baseline, and consent to participate. The inclusion criteria for the control group were adults aged 40–80, without IHD with the physician’s approval, and consent to participate. The exclusion criteria of the case and control groups were a history of mental disorders, cancer, malignant diseases, using lycopene supplements, and failure in gathering the required data.

The participant’s body weight was measured with clothing and without shoes and recorded to the nearest 0.1 kg using a digital scale. Their height was measured in a standing position without shoes and with a tapeline with an accuracy of 1 cm. Socio-demographic, medical, and dietary data were collected using a self-administered questionnaire consisted of three parts: first, general information such as age, gender, height, weight, and place of residence. Afterward, medical and lifestyle information including the use of medicine or supplements, smoking and physical was collected. Also, food intake was evaluated using a 237-item semi-quantitative food frequency questionnaire (FFQ) with standard portion sizes commonly consumed by Iranian people. The validity and reliability of FFQ was already confirmed in Iran for the evaluation of nutrients’ intake (31). Data on food intake during the last year in the control group and related to food intake in the last year before cancer diagnosis in the case group were collected through a face-to-face interviews by a trained dietitian. All reported consumptions were converted to grams per day by using household measures. Then, the intake of dietary lycopene was analyzed using Nutritionist IV software (version 7.0; N-Squared Computing, Salem, OR, USA). Data on biochemical and hematologic indices including red blood cells (RBC), white blood cells (RBC), fasting blood sugar (FBS), SBP (systolic blood pressure), right DBP (diastolic blood pressure), hemoglobin (HGB), hematocrit (HCT), mean corpuscular volume (MCV), mean corpuscular hemoglobin (MCH), the mean corpuscular hemoglobin concentration (MCHC), high-density lipoprotein cholesterol (HDLC), low-density lipoprotein cholesterol (LDLC), and triglycerides (TG) were extracted from participants’ file.

### Statistical analysis

An independent sample T-test (shown as mean ± sd), and chi-squared test [shown as frequency(percent)] were used for quantitative and qualitative data, respectively. Normal distribution of continuous data was confirmed using the Kolmogorov–Smirnov test. Logistic regression method [shown as OR and 95% Confidence Interval (CI)] was used for the association between IHD and dietary intake of lycopene and the confounding variables including age at interview, gender, total fat and energy intake, BMI, smoking, using alcohol, and physical activity were adjusted in different models. Data was performed using SPSS software version 21 (IBM Corp., Armonk, NY, USA) and *p* < 0.05 was considered significant in all analyses.

### Ethical considerations

The informed written consent was obtained from all participants. This study has been approved by Local ethics review boards at Shahid Beheshti University, Tehran, Iran (Code: IR.SBMU.NNFTRI.REC.1400.030).

## Results

Characteristics of the participants are presented in Table 1. No significant difference was found regarding age, sex, physical activity, height, weight, BMI, smoking, and drink alcohol between the groups. Table 2 presents the biochemical measurements of the participants. The cases had lower RBC (4.86 ± 1.66 vs. 4.96 ± 1.52, *p* < 0.01) and higher WBC (6.67 ± 0.53 vs. 6.32 ± 0.56, p < 0.01) and FBS (121.11 ± 37.36 vs. 107.96 ± 43.20, p < 0.01) than the controls. There was no significant difference in BMI, smoking, drink alcohol, right SBP, right DBP, HGB, HCT, MCV, MCH, MCHC, HDLC, LDLC, TG, and cholesterol.

**Table 1:** General characteristics of the participants.

	Cases (*n* = 443)	Controls (*n* = 443)	*p* value*
Age (y)	55.59 ± 14.43	54.67 ± 11.13	0.106
MET (kcal/kg*h)	37.53 ± 7.72	38.01 ± 8.38	0.391
Height (Cm)	161.48 ± 34.16	161.03 ± 27.61	0.461
Weight (Kg)	74.38 ± 17.31	72.64 ± 15.54	0.057
BMI (Kg/m^2^)	28.54 ± 6.3	28.06 ± 6.01	0.135
Smoking (n, %)	108 (24.83)	87 (19.21)	0.064
Male (n, %)	208 (47.92)	204 (45.03)	0.402
Drink Alcohol (n, %)	38 (8.74)	45 (9.94)	0.479

*Independent sample *T*-test (shown as mean ± sd) and chi-squared test [shown as frequency(percent)]. MET: metabolic equivalent of task, BMI: body mass index.

**Table 2:** Biochemical measurements of the participants.

	Cases (*n* = 443)	Controls (*n* = 443)	*p* value*
Right SBP (mmHg)	114.47 ± 16.63	114.51 ± 17.37	0.871
Right DBP (mmHg)	71.950 ± 10.40	71.96 ± 10.64	0.842
WBC (K/μL)	6.67 ± 0.53	6.32 ± 0.56	0.001
RBC (M/μL)	4.86 ± 1.66	4.96 ± 1.52	0.003
Hb (gr/dl)	13.99 ± 1.53	14.08 ± 1.55	0.428
HCT (%)	41.04 ± 4.14	41.36 ± 4.29	0.173
MCV (fL)	84.87 ± 5.81	85.002 ± 5.72	0.804
MCH (pg)	28.95 ± 2.59	28.97 ± 2.54	0.852
MCHC (gr)	34.08 ± 1.43	34.05 ± 1.41	0.164
PLT (K/μL)	283.75 ± 67.55	276.68 ± 68.09	0.204
FBS (mg/dl)	121.11 ± 37.36	107.96 ± 43.20	0.001
BUN (mg/dl)	13.58 ± 3.75	13.89 ± 3.82	0.094
Creatinine (mg/ml)	1.08 ± 0.27	1.10 ± 0.216	0.683
TG (mg/dl)	148.33 ± 109.08	144.51 ± 95.82	0.282
Cholesterol (mg/dl)	192.03 ± 40.39	191.26 ± 40.12	0.831
SGOT (IU/L)	19.97 ± 7.39	20.40 ± 10.05	0.091
SGPT (IU/L)	21.95 ± 13.52	22.29 ± 16.61	0.093
ALP (IU/L)	222.07 ± 68.79	222.21 ± 67.23	0.673
HDLC (mg/dl)	52.42 ± 10.54	52.35 ± 10.69	0.876
LDLC (mg/dl)	110.16 ± 34.06	110.40 ± 33.43	0.812

*Independent sample t-test. SBP: systolic blood pressure, DBP: diastolic blood pressure, WBC: white blood cell, RBC: red blood cell, FBS: fasting blood sugar, TG: triglyceride, HDL-c: high density lipoprotein cholesterol, LDL-c: low-density lipoprotein cholesterol.

A comparison of dietary intake among the case and control groups is presented in Table 3. The case group had a lower intake of lycopene (12.99 + 8.42 vs. 14.234 + 7.28 mg/d, *p* = 0.01) than the control group. No significant difference was found in dietary intake of protein, total fat, carbohydrate, energy, saturated fatty acids, and other micronutrients between the groups.

**Table 3:** Dietary nutrient intake among the Cases and the controls.

	Cases (*n* = 443)	Controls (*n* = 443)	*p* value*
Protein (g/day)	78.37 ± 25.76	78.97 ± 26.36	0.452
Fat (g/day)	64.39 ± 24.79	64.59 ± 25.71	0.228
Carbohydrate (g/day)	409.62 ± 135.34	415.49 ± 140.59	0.193
Calorie (Kcal/day)	2482.58 ± 768.69	2511.78 ± 799.43	0.254
Lycopene (mg/day)	12.99 ± 8.42	14.234 ± 7.28	0.011
Galactose (mg/day)	0.192 ± 0.188	0.21 ± 0.216	0.069
Fiber (g/day)	27.11 ± 10.37	28.01 ± 10.12	0.224
Calcium (mg/day)	912.24 ± 328.5	918.67 ± 329.5	0.814
Iron (mg/day)	13.38 ± 4.73	344.82 ± 110.76	0.801
Magnesium (mg/day)	344.8 ± 110.76	343.98 ± 110.69	0.835
Phosphorus (mg/day)	1198.2 ± 389.77	1199.44 ± 398.51	0.417
Potassium (mg/day)	3674.27 ± 1278.79	3642.93 ± 1257.57	0.744
Sodium (mg/day)	4554.7 ± 2054.61	4633.84 ± 2088.36	0.788
Zinc (mg/day)	10.08 ± 3.35	10.08 ± 3.42	0.438
Copper (mg/day)	1.83 ± 0.71	1.83 ± 0.66	0.662
Fluoride (mg/day)	3573.2 ± 2394.6	3576.44 ± 2342.34	0.231
Manganese (mg/day)	5.65 ± 1.95	5.66 ± 1.93	0.916
Selenium (μg/day)	55.78 ± 29.63	54.66 ± 28.13	0.658
Vitamin A (IU/d)	8692.01 ± 5640.81	8720.66 ± 5552.12	0.621
Retinol (IU/d)	338.25 ± 394.44	333.88 ± 281.89	0.553
Vitamin A (mg/day)	715.08 ± 508.06	713.19 ± 425.37	0.964
Beta Carotene (μg/day)	4019.38 ± 2739.06	4044.64 ± 2746.80	0.411
Alpha Carotene (μg/day)	665.44 ± 838.02	685.43 ± 856.74	0.206
Alpha tocopherol (mg/day)	7.24 ± 3.28	7.14 ± 3.22	0.654
Vitamin D (IU)	42.91 ± 27.26	42.96 ± 27.10	0.291
Vitamin D2, D3 (mg/day)	1.21 ± 0.703	1.21 ± 0.69	0.239
Vitamin C (mg/day)	143.73 ± 83.78	139.96 ± 78.97	0.448
Vitamin B1 (mg/day)	1.62 ± 0.59	1.64 ± 0.60	0.821
Vitamin B2 (mg/day)	1.76 ± 0.66	1.74 ± 0.626	0.544
Vitamin B3 (mg/day)	18.16 ± 6.53	18.17 ± 6.51	0.682
Vitamin B5 (mg/day)	5.93 ± 1.90	5.90 ± 1.92	0.452
Vitamin B6 (mg/day)	9.77 ± 5.91	10.04 ± 4.91	0.511
Folate (μg/day)	381.07 ± 138.89	377.18 ± 130.77	0.358
Vitamin B12 (μg/day)	6.07 ± 6.43	5.87 ± 4.50	0.257
Vitamin K (mg/day)	164.5 ± 102.65	165.39 ± 100.9	0.542

*Independent sample *t*-test.

The association of IHD and dietary intake of lycopene is presented in Table 4. A negative association was found between IHD and lycopene (OR: 0.98, CI 95%: 0.963–0.99, *p* = 0.021) (Model 1). The results remained significant after adjustment for age and sex (OR: 0.980, CI 95%: 0.96–0.99, *p* = 0.024) (Model 2), after additional adjustment for dietary calorie and total fat (OR: 0.98, CI 95%: 0.96–0.99, p = 0.024) (Model 3), after further adjustments for BMI (OR: 0.97, CI 95%: 0.96–0.99, *p* = 0.016) (Model 4), and after further adjustments for smoking, drink alcohol, and and physical activity (OR: 0.97, CI 95%: 0.95–0.99, *p* = 0.015) (Model 5). The IHD relationship with the categorical values of the lycopene was also evaluated. The risk of IHD in people with the highest quartile of dietary intake of lycopene was significantly lower than those with the lowest quartile (OR = 0.67, CI 95%: 0.46–0.97, *p* = 0.036). This association remained significant after adjusting the confounders (Table 4).

**Table 4:** Odds ratio and CI95% of the association between ischemic heart disease (IHD) and dietary intake of lycopene.

	Trend	Quartile 1 (<7.91 mg/d)	Quartile 2 (7.91–11.64 mg/d)	Quartile 3 (11.64–16.47 mg/d/)	Quartile 4 (16.47 < mg/d)
Model 1	**0.98 (0.96–0.99)**	**1**	**0.79 (0.54–1.15)**	**1.14 (0.78–1.66)**	**0.67 (0.46–0.97)**
Model 2	0.98 (0.96–0.99)	1	0.79 (0.54–1.16)	1.13 (0.77–1.65)	0.67 (0.46–0.99)
Model 3	0.98 (0.96–0.99)	1	0.78 (0.53–1.15)	1.11 (0.75–1.64)	0.65 (0.42–0.99)
Model 4	0.97 (0.96–0.99)	1	0.77 (0.52–1.13)	1.09 (0.73–1.61)	0.63 (0.41–0.97)
Model 5	0.97 (0.95–0.99)	1	0.78 (0.53–1.15)	1.07 (0.72–1.59)	0.64 (0.41–0.99)

* Binominal Logistic regression, Model 1: crude, Model 2: adjusted for Age at interview and gender, Model 3: Additionally adjusted for total fat and energy, Model 4: Additionally adjusted for BMI, Model 5: Further adjusting for smoking, drink alcohol, and physical activity.

## Discussion

**Figure 1:**
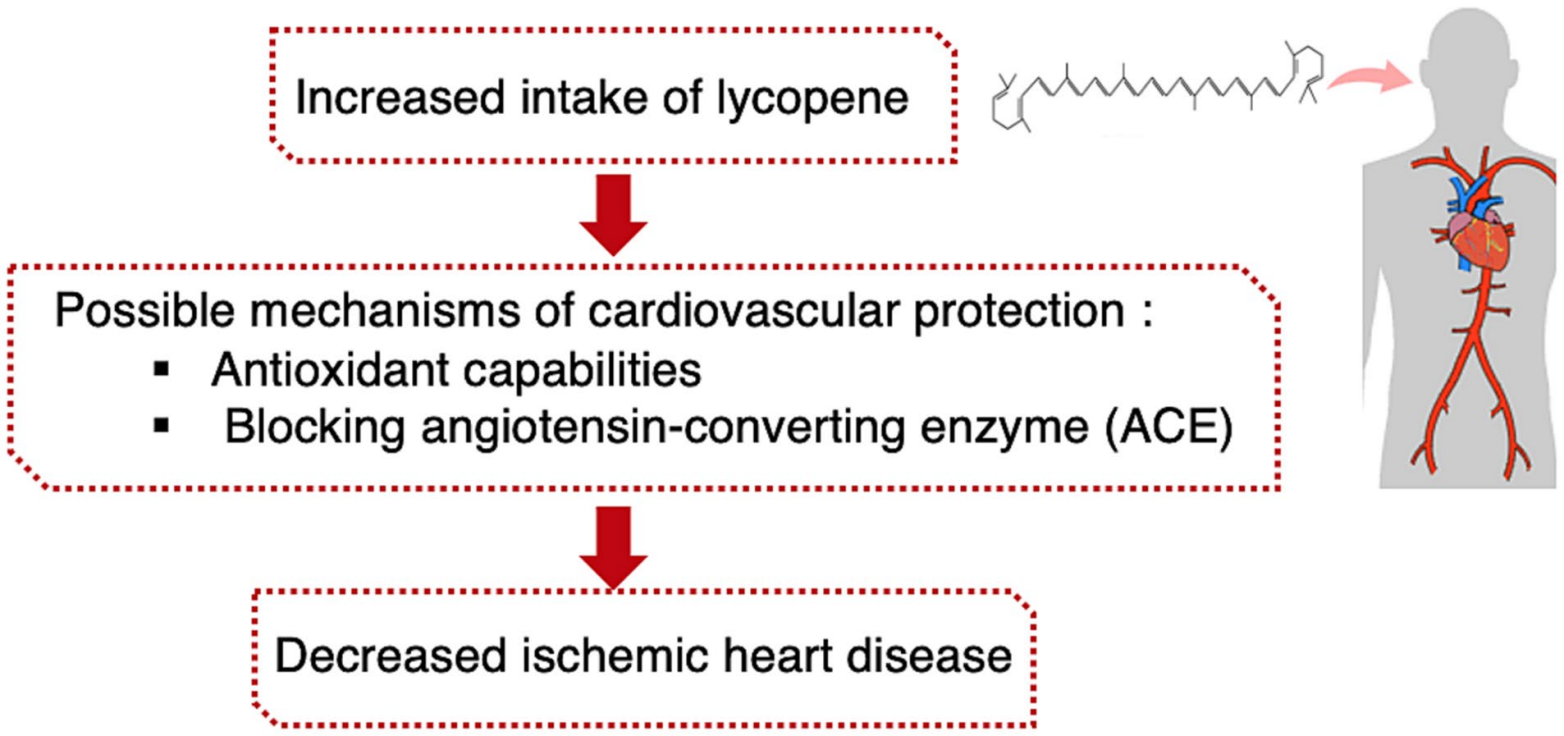
A significant negative association was found between intake of lycopene and ischemic heart disease.

According to this case–control study, the patients with IHD had a lower lycopene intake than the control group. The present study discovered an inverse association between lycopene intake and the risk of IHD. The associations remained significant after age and sex, after additional adjustments for dietary calorie and total fat, after additional adjustments for BMI, and after further adjustments for smoking and physical activity (Figure 1). In line with the present findings, a population-based study has shown that a lower risk for acute coronary events or stroke was associated with higher serum lycopene concentration (13). Data from previous studies suggests that consuming more lycopene-containing foods leads to higher levels of lycopene in the bloodstream (32). Moreover, high serum levels of lycopene were significantly related to low hazard ratios for CVD mortality in a Japanese population-based study (33). In addition, Rissanen et al. demonstrated that a low plasma concentration of lycopene was associated with a 17.8% increase in the carotid intima-media thickness (CIMT) in men compared to subjects with higher plasma concentrations of lycopene after adjustments for cardiovascular risk factors and nutrients intake (34). In addition, a cross-sectional study on 1,028 middle-aged men confirmed that low serum lycopene concentrations were associated with higher CIMT in middle-aged men (35). On the other hand, another study by Bruneck et al. found no association between lycopene plasma levels and atherosclerosis (36). Moreover, a nested case–control study utilizing the PHS database did not find any association between increasing concentrations of plasma lycopene and the risk of CVD (37). It is important to note that the conflicting results on the potential cardioprotective effects of lycopene may be caused by the wide variety of experimental protocols used to discover the association between lycopene consumption and cardiovascular disease (38). Pre-existing levels of lycopene, the dietary source of lycopene, and the characteristics of the target populations are essential factors that can affect any association between lycopene consumption and cardiovascular disease (38).

Possible explanations for the effect of lycopene on IHD might be the antithrombotic and antiplatelet effects of lycopene (39, 40), potent antioxidant properties of lycopene (41, 42), induction of detoxifying enzymes (43, 44) and reduction of cell surface adhesion and intima-media thickness (45). Oxidative stress can lead to the production of proinflammatory mediators, including vascular cell adhesion molecules, intracellular adhesion molecules, and chemoattractant proteins, which contribute to the development of early atherosclerosis (46, 47). On the other hand, lycopene is a powerful antioxidant that can effectively reduce levels of reactive oxygen species and eliminate singlet oxygen (48, 49). Thus, lycopene may suppress oxidative stress and acts against IHD. The strength of the present study is the adjustment for a broad range of potential confounding factors. However, this study had some limitations. First, the study design was case–control and did not allow to discover the cause and effect relationship. Second, the FFQ was used to assess food intake in the study, which may lead to over-reporting or under-reporting of dietary intake. Third, the way of cooking food was not investigated in the present study, which can affect the bioavailability of food lycopene.

## Conclusion

A significant negative association was found between intake of lycopene and IHD. If this result is confirmed in future studies, high dietary intake of lycopene and lycopene supplementation can be considered complementary strategies against IHD. Further prospective studies in different populations are required to elucidate the roles of lycopene against IHD.

## Data availabiulity statement

The raw data supporting the conclusions of this article will be made available by the authors, without undue reservation.

## Ethics statement

The ethics committee of Shahid Beheshti University of Medical Sciences, Tehran, Iran approved the study (Ethics Code: IR.SBMU.nnftri.Rec.1400.030). The study was conducted in accordance with the local legislation and institutional requirements. The participants provided their written informed consent to participate in this study.

## Author contributions

SD: Formal analysis, Writing – original draft. AA: Data curation, Writing – review & editing. FAl: Data curation, Writing – original draft. MM: Software, Writing – review & editing. RM: Software, Writing – review & editing. FAz: Software, Writing – review & editing. AR: Methodology, Writing – review & editing. ME: Formal analysis, Writing – review & editing. SS: Software, Writing – review & editing. MA: Data curation, Writing – review & editing. SK: Data curation, Writing – review & editing. MA: Data curation, Writing – review & editing. MG: Writing – original draft, Writing – review & editing.

## References

[1] Jensen RV Hjortbak MV Bøtker HE. Ischemic heart disease: an update. InSeminars in nuclear medicine (2020). WB Saunders. 50, 195–207. 10.1053/j.semnuclmed.2020.02.007 32284106

[2] Khan MA Hashim MJ Mustafa H Baniyas MY Al Suwaidi SKBM AlKatheeri R. Global epidemiology of ischemic heart disease: results from the global burden of disease study. Cureus. (2020) 12. doi: 10.7759/cureus.9349 PMC7384703 32742886

[3] Flynn CD Morris P Manuel L Matteucci M Ronco D Massimi G. Systematic review and meta-analysis of the mechanical complications of ischemic heart disease: papillary muscle rupture, left ventricle rupture and post-infarct ventricular septal defect. Annals of Cardiothoracic Surgery. (2022) 11:195–209. doi: 10.21037/acs-2022-ami-24, PMID: 35733707 PMC9207690

[4] Damluji AA Van Diepen S Katz JN Menon V Tamis-Holland JE Bakitas M. Mechanical complications of acute myocardial infarction: a scientific statement from the American Heart Association. Circulation. (2021) 144:e16–35. doi: 10.1161/CIR.0000000000000985, PMID: 34126755 PMC9364424

[5] Sarrafzadegan N Mohammmadifard N. Cardiovascular disease in Iran in the last 40 years: prevalence, mortality, morbidity, challenges and strategies for cardiovascular prevention. Arch Iran Med. (2019) 22:204–10. PMID: 31126179

[6] Gupta R Wood DA. Primary prevention of ischaemic heart disease: populations, individuals, and health professionals. Lancet. (2019) 394:685–96. doi: 10.1016/S0140-6736(19)31893-8, PMID: 31448740

[7] Hertog MG Feskens EJ Kromhout D. Antioxidant flavonols and coronary heart disease risk. Lancet. (1997) 349:699. doi: 10.1016/S0140-6736(05)60135-3 9078206

[8] Wu Y Qian Y Pan Y Li P Yang J Ye X. Association between dietary fiber intake and risk of coronary heart disease: a meta-analysis. Clin Nutr. (2015) 34:603–11. doi: 10.1016/j.clnu.2014.05.009, PMID: 24929874

[9] Kobylecki CJ Afzal S Davey Smith G Nordestgaard BG. Genetically high plasma vitamin C, intake of fruit and vegetables, and risk of ischemic heart disease and all-cause mortality: a Mendelian randomization study. Am J Clin Nutr. (2015) 101:1135–43. doi: 10.3945/ajcn.114.104497, PMID: 25948669

[10] He FJ MacGregor GA. Beneficial effects of potassium. BMJ. (2001) 323:497–501. doi: 10.1136/bmj.323.7311.497, PMID: 11532846 PMC1121081

[11] Miller ER III Appel LJ Risby TH. Effect of dietary patterns on measures of lipid peroxidation: results from a randomized clinical trial. Circulation. (1998) 98:2390–5. doi: 10.1161/01.CIR.98.22.2390 9832483

[12] Ros E Hu FB. Consumption of plant seeds and cardiovascular health: epidemiological and clinical trial evidence. Circulation. (2013) 128:553–65. doi: 10.1161/CIRCULATIONAHA.112.001119, PMID: 23897849 PMC3745769

[13] Mordente A Guantario B Meucci E Silvestrini A Lombardi E Martorana GE. Lycopene and cardiovascular diseases: an update. Curr Med Chem. (2011) 18:1146–63. doi: 10.2174/092986711795029717, PMID: 21291369

[14] Jackson H Braun CL Ernst H. The chemistry of novel xanthophyll carotenoids. Am J Cardiol. (2008) 101:S50–7. doi: 10.1016/j.amjcard.2008.02.008 18474275

[15] Rao AV Ray M Rao L. Lycopene. Adv Food Nutr Res. (2006) 51:99–164. doi: 10.1016/S1043-4526(06)51002-2 17011475

[16] Kong K-W Khoo H-E Prasad KN Ismail A Tan C-P Rajab NF. Revealing the power of the natural red pigment lycopene. Molecules. (2010) 15:959–87. doi: 10.3390/molecules15020959, PMID: 20335956 PMC6263198

[17] Bahonar A Saadatnia M Khorvash F Maracy M Khosravi A. Carotenoids as potential antioxidant agents in stroke prevention: a systematic review. Int J Prev Med. (2017) 8:70. doi: 10.4103/ijpvm.IJPVM_112_17 28983399 PMC5625359

[18] Bin-Jumah MN Nadeem MS Gilani SJ Mubeen B Ullah I Alzarea SI. Lycopene: a natural arsenal in the war against oxidative stress and cardiovascular diseases. Antioxidants. (2022) 11:232. doi: 10.3390/antiox11020232, PMID: 35204115 PMC8868303

[19] Karimian B Soleimani A Mohammadsharifi G Heshmat-Ghahdarijani K Rejali L Shafie D. Effect of lycopene supplementation on some cardiovascular risk factors and markers of endothelial function in Iranian patients with ischemic heart failure: a randomized clinical trial. Cardiol Res Pract. (2022) 2022:1–7. doi: 10.1155/2022/2610145, PMID: 36337273 PMC9635958

[20] Aune D Giovannucci E Boffetta P Fadnes LT Keum N Norat T. Fruit and vegetable intake and the risk of cardiovascular disease, total cancer and all-cause mortality—a systematic review and dose-response meta-analysis of prospective studies. Int J Epidemiol. (2017) 46:1029–56. doi: 10.1093/ije/dyw319, PMID: 28338764 PMC5837313

[21] Appel LJ Moore TJ Obarzanek E Vollmer WM Svetkey LP Sacks FM. A clinical trial of the effects of dietary patterns on blood pressure. N Engl J Med. (1997) 336:1117–24. doi: 10.1056/NEJM199704173361601 9099655

[22] John J Ziebland S Yudkin P Roe L Neil H. Effects of fruit and vegetable consumption on plasma antioxidant concentrations and blood pressure: a randomised controlled trial. Lancet. (2002) 359:1969–74. doi: 10.1016/S0140-6736(02)98858-6 12076551

[23] Svendsen M Blomhoff R Holme I Tonstad S. The effect of an increased intake of vegetables and fruit on weight loss, blood pressure and antioxidant defense in subjects with sleep related breathing disorders. Eur J Clin Nutr. (2007) 61:1301–11. doi: 10.1038/sj.ejcn.1602652 17268408

[24] Sesso HD Buring JE Norkus EP Gaziano JM. Plasma lycopene, other carotenoids, and retinol and the risk of cardiovascular disease in women. Am J Clin Nutr. (2004) 79:47–53. doi: 10.1093/ajcn/79.1.47, PMID: 14684396

[25] Street DA Comstock GW Salkeld RM Schüep W Klag MJ. Serum antioxidants and myocardial infarction. Are low levels of carotenoids and alpha-tocopherol risk factors for myocardial infarction? Circulation. (1994) 90:1154–61. doi: 10.1161/01.CIR.90.3.1154, PMID: 8087925

[26] Kohlmeier L Kark JD Gomez-Gracia E Martin BC Steck SE Kardinaal AF. Lycopene and myocardial infarction risk in the EURAMIC study. Am J Epidemiol. (1997) 146:618–26. doi: 10.1093/oxfordjournals.aje.a009327, PMID: 9345115

[27] Howard A Williams N Palmer C Cambou J Evans A Foote J. Do hydroxy-carotenoids prevent coronary heart disease? A comparison between Belfast and Toulouse. International Journal for Vitamin and Nutrition research Internationale Zeitschrift fur Vitamin-und Ernahrungsforschung Journal International de Vitaminologie et de Nutrition. (1996) 66:113–8. PMID: 8843985

[28] Klipstein-Grobusch K Launer L Geleijnse J Boeing H Hofman A Witteman J. Serum carotenoids and atherosclerosis: the Rotterdam study. Atherosclerosis. (2000) 148:49–56. doi: 10.1016/S0021-9150(99)00221-X 10580170

[29] Song B Liu K Gao Y Zhao L Fang H Li Y. Lycopene and risk of cardiovascular diseases: a meta-analysis of observational studies. Mol Nutr Food Res. (2017) 61:1601009. doi: 10.1002/mnfr.201601009 28318092

[30] Sullivan KM Dean A Soe MM. OpenEpi: A web-based epidemiologic and statistical calculator for public health. Public health reports. (2009) 124:471–4. doi: 10.1177/003335490912400320 19445426 PMC2663701

[31] Mirmiran P Esfahani FH Mehrabi Y Hedayati M Azizi F. Reliability and relative validity of an FFQ for nutrients in the Tehran lipid and glucose study. Public Health Nutr. (2010) 13:654–62. doi: 10.1017/S1368980009991698, PMID: 19807937

[32] Biddle MJ Lennie TA Bricker GV Kopec RE Schwartz SJ Moser DK. Lycopene dietary intervention: a pilot study in patients with heart failure. J Cardiovasc Nurs. (2015) 30:205–12. doi: 10.1097/JCN.0000000000000108, PMID: 24651682 PMC4168010

[33] Ito Y Kurata M Suzuki K Hamajima N Hishida H Aoki K. Cardiovascular disease mortality and serum carotenoid levels: a Japanese population-based follow-up study. J Epidemiol. (2006) 16:154–60. doi: 10.2188/jea.16.154, PMID: 16837766 PMC7603911

[34] Rissanen T Voutilainen S Nyyssönen K Salonen R Salonen JT. Low plasma lycopene concentration is associated with increased intima-media thickness of the carotid artery wall. Arterioscler Thromb Vasc Biol. (2000) 20:2677–81. doi: 10.1161/01.ATV.20.12.2677, PMID: 11116071

[35] Rissanen TH Voutilainen S Nyyssönen K Salonen R Kaplan GA Salonen JT. Serum lycopene concentrations and carotid atherosclerosis: the Kuopio ischaemic heart disease risk factor study. Am J Clin Nutr. (2003) 77:133–8. doi: 10.1093/ajcn/77.1.133, PMID: 12499332

[36] D'Odorico A Martines D Kiechl S Egger G Oberhollenzer F Bonvicini P. High plasma levels of α-and β-carotene are associated with a lower risk of atherosclerosis: results from the Bruneck study. Atherosclerosis. (2000) 153:231–9. doi: 10.1016/S0021-9150(00)00403-2, PMID: 11058719

[37] Sesso HD Buring JE Norkus EP Gaziano JM. Plasma lycopene, other carotenoids, and retinol and the risk of cardiovascular disease in men. Am J Clin Nutr. (2005) 81:990–7. doi: 10.1093/ajcn/81.5.990, PMID: 15883420

[38] Costa-Rodrigues J Pinho O Monteiro P. Can lycopene be considered an effective protection against cardiovascular disease? Food Chem. (2018) 245:1148–53. doi: 10.1016/j.foodchem.2017.11.055, PMID: 29287334

[39] O’Kennedy N Crosbie L Whelan S Luther V Horgan G Broom JI. Effects of tomato extract on platelet function: a double-blinded crossover study in healthy humans. Am J Clin Nutr. (2006) 84:561–9. doi: 10.1093/ajcn/84.3.561, PMID: 16960170

[40] O’Kennedy N Crosbie L van Lieshout M Broom JI Webb DJ Duttaroy AK. Effects of antiplatelet components of tomato extract on platelet function in vitro and ex vivo: a time-course cannulation study in healthy humans. Am J Clin Nutr. (2006) 84:570–9. doi: 10.1093/ajcn/84.3.570, PMID: 16960171

[41] Heber D Lu Q-Y. Overview of mechanisms of action of lycopene. Exp Biol Med. (2002) 227:920–3. doi: 10.1177/153537020222701013, PMID: 12424335

[42] Di Mascio P Kaiser S Sies H. Lycopene as the most efficient biological carotenoid singlet oxygen quencher. Arch Biochem Biophys. (1989) 274:532–8. doi: 10.1016/0003-9861(89)90467-0, PMID: 2802626

[43] Zaripheh S Nara TY Nakamura MT Erdman JW Jr. Dietary lycopene downregulates carotenoid 15, 15′-monooxygenase and PPAR-γ in selected rat tissues. J Nutr. (2006) 136:932–8. doi: 10.1093/jn/136.4.932, PMID: 16549453

[44] Dingley KH Ubick EA Chiarappa-Zucca ML Nowell S Abel S Ebeler SE. Effect of dietary constituents with chemopreventive potential on adduct formation of a low dose of the heterocyclic amines PhIP and IQ and phase II hepatic enzymes. Nutr Cancer. (2003) 46:212–21. doi: 10.1207/S15327914NC4602_15, PMID: 14690798

[45] Denniss SG Haffner TD Kroetsch JT Davidson SR Rush JW Hughson RL. Effect of short-term lycopene supplementation and postprandial dyslipidemia on plasma antioxidants and biomarkers of endothelial health in young, healthy individuals. Vasc Health Risk Manag. (2008) 4:213–22. doi: 10.2147/vhrm.2008.04.01.213, PMID: 18629373 PMC2464768

[46] Dzau VJ Antman EM Black HR Hayes DL Manson JE Plutzky J. The cardiovascular disease continuum validated: clinical evidence of improved patient outcomes: part I: pathophysiology and clinical trial evidence (risk factors through stable coronary artery disease). Circulation. (2006) 114:2850–70. doi: 10.1161/CIRCULATIONAHA.106.655688, PMID: 17179034

[47] Koenen RR Weber C. Therapeutic targeting of chemokine interactions in atherosclerosis. Nat Rev Drug Discov. (2010) 9:141–53. doi: 10.1038/nrd3048 20118962

[48] Joshi B Kar SK Yadav PK Yadav S Shrestha L Bera TK. Therapeutic and medicinal uses of lycopene. A systematic review. (2020) 8:1195. doi: 10.18203/2320-6012.ijrms20200804

[49] Khan UM Sevindik M Zarrabi A Nami M Ozdemir B Kaplan DN. Lycopene: food sources, biological activities, and human health benefits. Oxidative Med Cell Longev. (2021) 2021:1–10. doi: 10.1155/2021/2713511, PMID: 34840666 PMC8626194

